# FixingTIM: interactive exploration of sequence and structural data to identify functional mutations in protein families

**DOI:** 10.1186/1753-6561-8-S2-S3

**Published:** 2014-08-28

**Authors:** Timothy Luciani, John Wenskovitch, Koonwah Chen, David Koes, Timothy Travers, G Elisabeta Marai

**Affiliations:** 1 Department of Computer Science, Brown University, Box 1910, 02912 Providence, RI, US; 2 Department of Computer Science, University of Pittsburgh, 210 South Bouquet, 15260 Pittsburgh, PA, US; 3 School of Information Science, University of Pittsburgh, 135 North Bellefield Avenue, 15260 Pittsburgh, PA, US; 4 Department of Computational and Systems Biology, University of Pittsburgh, 3501 Fifth Avenue, 15260 Pittsburgh, PA, US; 5 Department of Computer Science, University of Illinois at Chicago, 851 S. Morgan St., 60607 Chicago, IL, US

## Abstract

**Background:**

Knowledge of the 3D structure and functionality of proteins can lead to insight into the associated cellular processes, speed up the creation of pharmaceutical products, and develop drugs that are more effective in combating disease.

**Methods:**

We present the design and implementation of a visual mining and analysis tool to help identify protein mutations across a family of structural models and to help discover the effect of these mutations on protein function. We integrate 3D structure and sequence information in a common visual interface; multiple linked views and a computational backbone allow comparison at the molecular and atomic levels, while a novel trend-image visual abstraction allows for the sorting and mining of large collections of sequences and of their residues.

**Results:**

We evaluate our approach on the triosephosphate isomerase (TIM) family structural models and sequence data and show that our tool provides an effective, scalable way to navigate a family of proteins, as well as a means to inspect the structure and sequence of individual proteins.

**Conclusions:**

The TIM application shows that our tool can assist in the navigation of families of proteins, as well as in the exploration of individual protein structures. In conjunction with domain expert knowledge, this interactive tool can help provide biophysical insight into why specific mutations affect function and potentially suggest additional modifications to the protein that could be used to rescue functionality.

## Background

By determining the 3D structure and functionality of proteins, biologists can gain insight into the associated cellular processes, speed up the creation of pharmaceutical products, and develop drugs that are more effective in combating disease. A variety of protein-sequencing techniques are currently available; these techniques enable biologists to examine amino acid sequences. As amino acid sequence ultimately determines protein 3D structure, mining of sequence information may facilitate the discovery of correlations between protein structure and functionality. However, the vast number of proteins sequenced by scientists make interactive mining tools necessary in solving this problem.

To improve the exploration process, many efforts have been made, from folding the sequences through classification [1,2], to tools for 3D view exploration [3] and to web-based applications which present large amounts of information to the users [4]. Nevertheless, challenges in solving this mining problem remain, from addressing scalability to spatial and non-spatial data integration and to tool integration.

**Figure 1:**
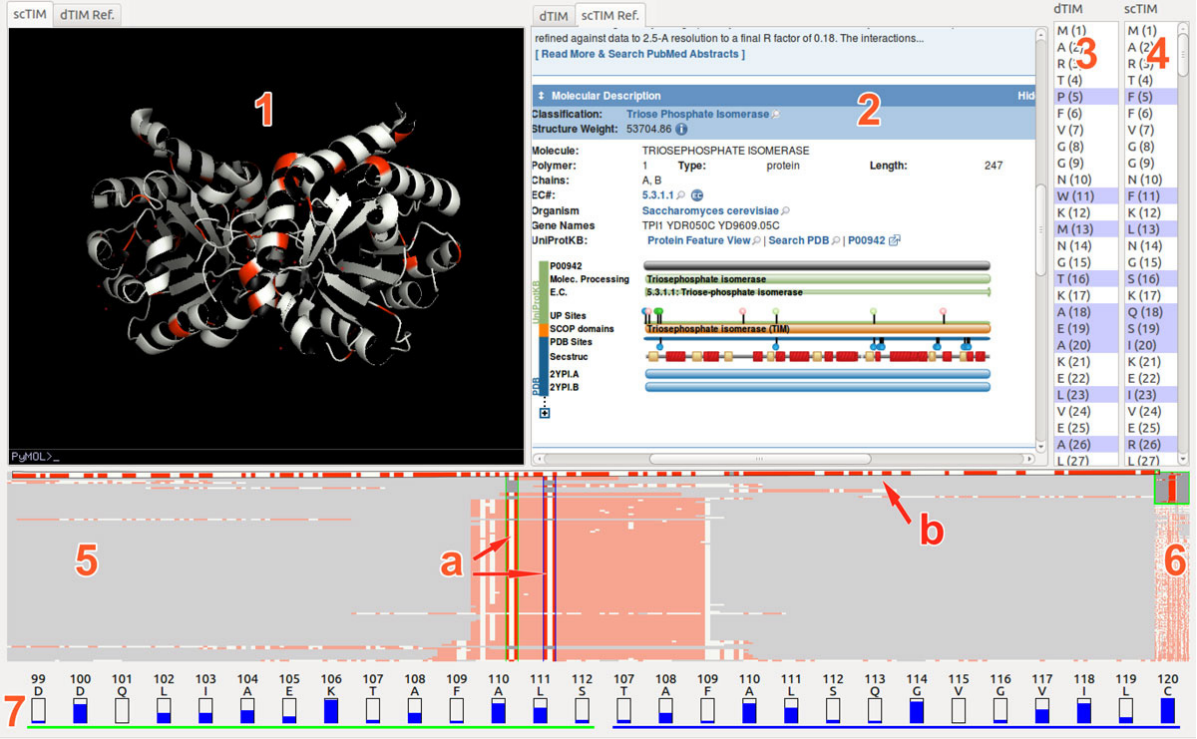
**FixingTIM visual interface with four panels: a 3D view and reference information panel (1 and 2); a protein sequence viewer (3 and 4); a trend image panel for aggregating protein families (5 and 6) with two fragment paddles (5a) and a sequence paddle (5b); and a residue view for residue distribution information (7)**.

We introduce a novel visualization tool, FixingTIM (Figure 1), to help identify protein mutations across families of structural models, and to help discover the effect of these mutations on protein function. Following a rigorous data and task analysis, we pursue a client-server approach in which distributed data sources for 3D structure and non-spatial sequence information are integrated. To better address scalability concerns, we aggregate family-sequence data into a novel interactive pixel-based abstraction called a trend image. Interactive exploration, multiple linked views, and details on demand further allow the generation of hypotheses regarding structure and functionality correlations in a diverse and fragmented space. The tool is open source; a Linux distribution is publicly available at http://visualizlab.org/fixingTIM.

## Methods

### Data and task analysis

The design of our tool is informed by our domain data and task analysis. The data we consider in this application consists primarily of protein characteristics, where protein characteristics include structural information and amino acid sequence information. This data can be used to build a 3D representation of the protein that allows visualization of the atoms that comprise each residue in the protein as well as the bonds between these atoms.

The *protein structure*--determined theoretically or experimentally--is typically stored in a PDB file [5], alongside references to the studies that determined the structure of the proteins, the residue sequence (the sequence of amino acids that make up the protein), and the positions of each atom in 3D space. The structural data can be visually mapped to a 3D representation of the protein, which includes atoms, bonds, amino acids and protein chains. The *amino acid sequence *of each protein is typically stored in remote databases, for example, Uniprot [6]. Each sequence consists of a string of capital letters, each letter representing an amino acid (also called a residue) in the protein. To find regions of conservation within sequences belonging to the same protein family, these sequences can be aligned using a host of computational alignment tools, with gaps introduced to better align common subsequences present across the family. A particular sequence family may include special mutations, some functionally-defective. Finally, external web services [6] may provide additional relevant *metadata and data*, such as model-quality ratings provided by domain experts.

From the desirable features of a visual mining system (as indicated by the BioVis 2013 Data Contest), we focus on the following tasks:

- *Generate 3D protein structures *from sequence data

- *Inspect 3D *protein structures

- *Link to *online resources

- *Compare a single protein *to the rest of its family

- *Identify sequence mutation locations *on a family of proteins

- *Examine multiple sequence alignments*

- *Highlight specific residue locations *on the 3D protein structures

- *Examine residue distribution *across a protein family

### Client-server framework

**Figure 2:**
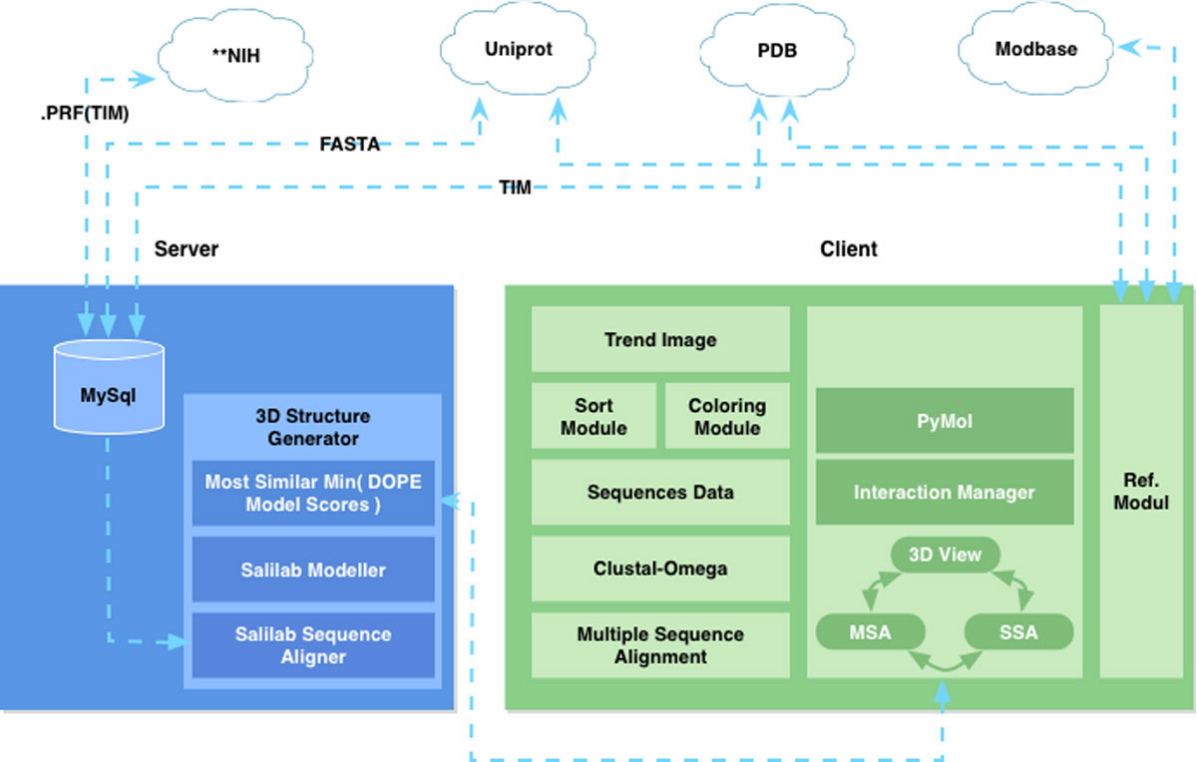
**Client-server architecture diagram, with TIM-instantiation as an example application**.

Given the variety and distributed nature of relevant domain data, we design and implement an overall client-server architecture (Figure 2). Our server fetches and caches in a local MySQL database the protein sequences, alignment, and 3D structures from ModBase, Uniprot, and the NIH BLAST server [7]. The server provides sequence and 3D structure data to the client. If a 3D structure does not exist for the protein, the server computes an approximate model using the Sali Lab Modeller toolkit [8].

Our client implements three core modules: a trend image module for exploring protein families; an interaction manager for viewing; and an external reference module for access to online catalogues. The three modules are linked, allowing for simultaneous interaction with the data in each abstraction.

The back-end of the tool is implemented in Python, C, and MySQL. The frontend of the tool uses Python as the primary development language, with Qt for the GUI and the PyMOL Molecular Graphics System [9] for rendering the protein structures.

**Figure 3:**
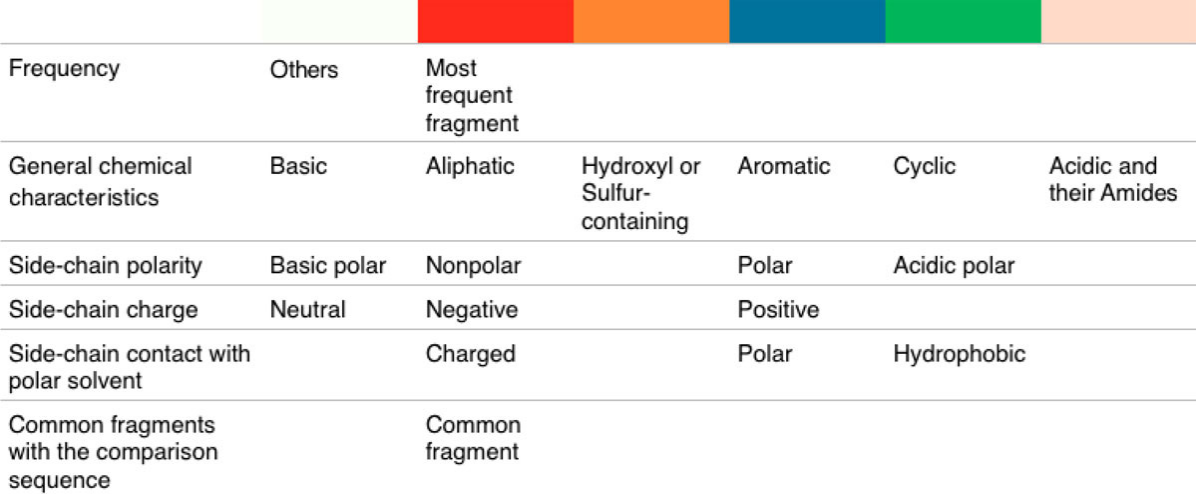
**Trend image coloring scheme based on residue properties and frequence of comparison metrics**.

### Visual design

Given the diversity and complementary nature of the data required, as well as the comparison nature of the domain-specific interactions, we pursue a linked multi-view top design. The visual interface consists of four linked panels (Figure 1): a tabbed 3D structure and reference viewing panel (two side-by-side views); a side-by-side protein sequence viewer; a trend image panel for exploring protein families; and a residue view for residue distribution information.

In this top design, the trend image view serves as the main anchor point of the interface. From this view, users can explore an entire protein family, and view the differences between family members. By right-clicking on a trend line, the user has the option of opening the structure file for this model in the 3D View, to compare it side-by-side with another model. Below, we describe each module in detail.

**Trend image panel**. The trend image view provides the ability to navigate and sort through large numbers of sequences. The trend-image is a pixel-based visual abstraction, in which each line represents the residues of a single protein sequence: each pixel row in the trend image corresponds to an individual protein sequence in the family. The trend image summarizes an entire protein family, aligned by one of several sorting algorithms, and colored by one of several different color schemes (e.g., Figure 3). The total width of the trend image corresponds to the longest protein sequence that is being aligned. Shorter protein sequences in the family have filler space, usually gray, inserted at the beginning and end of the alignment, so that they match the total width of the trend image. The x-axis indicates the position in the aligned sequences: each particular × corresponds to a residue on the protein.

The trend image view contains paddles for the selection of subsequences from a full family of protein sequences. These paddles also link to the residue distribution view at the bottom of the tool; this panel displays information about the distribution of amino acids, namely the fraction of proteins in the family that share the same residue as the selected protein.

A vertical overview pane (component 6 in Figure 1) provides a high-level view of the full dataset; while the two *fragment*-selection paddles allow narrowing the section of sequence considered for analysis and drilling for details. The horizontal sequence-selection paddle allows users to select a particular sequence. A selection event prompts the application to search for the 3D structure from online repositories; the 3D structure is presented if it already exists or it is generated on the fly if it does not.

We note that an earlier version of the software included a single fragment-selection paddle. However, upon repairing dTIM, the BioVis domain experts discovered two symmetric sequence pairings with identical mutations; once repaired, the function of the entire protein was restored. To identify these unique mutations required the ability to examine two parts of the sequence in detail, simultaneously. Based on this information, we implemented two vertical selection paddles in the trend image. These paddles allow for the examination of two locations in the sequence for residue conservation simultaneously, which alleviates the burden of individual inspection to identify symmetric pairings of mutations.

To facilitate navigation of the trend image, we provide a set of sorting algorithms. The sorting algorithms calculate a weight for each sequence relative to one input member of a protein family, and then order the sequences by their respective weights. We provide sorting by using the following measures as weights: fragment frequency; edit distance; weighted edit distance; number or percentage of common residues; number or percentage of common residues without regard to sequence position; number of residue subsequences of length N in common; and edit distance on selected residues.

In addition to the sorting algorithms, we also provide a ColorBrewer [10] set of coloring schemes to highlight a subset of the residues of each sequence. In each color scheme, black is used for residues that are not included in the scheme, whereas white is used to represent spacing in the sequence alignment. The residues in each internal class are given the same color. The list of coloring schemes is as follows: fragment frequency; general chemical characteristics; side-chain polarity; side-chain charge; and side-chain contact with polar solvent.

**Residue viewer**. The residue distribution view displays the fragment ID, fragment name, and the percentage of each residue type found in the same column (corresponding fragment in each sequence) for all sequences.

**3D viewer**. The top left panel of the tool provides two 3D structural views--one for the target protein, and one for the source protein, as well as a tabbed reference tool providing information about each protein. The structures can be examined at both the amino acid sequence level and at the atomic level. Through panning, zooming, rotation and details-on-demand operations (synchronized between the two views), users can observe different aspects of the two 3D structures.

Alternatively, the tabbed reference viewer allows users to access information from three complementary online data repositories: Uniprot, ModBase [11], and the RCSB Protein Data Bank. ModBase, for example, provides links to other databases, as well as ribbon diagrams for various models in the current sequence, and quality-criteria quantifying the reliability of certain model aspects.

**Protein sequence viewer**. To link in sequence information, the sequence panel lists the residue sequences for the selected structural models, with the differences between the two sequences highlighted in blue. The residues are selectable, and the selections are reflected in the 3D structure view.

## Results and discussion

We demonstrate our tool on a TIM protein-family application. These proteins play an important role in efficient energy production and can be found in nearly every organism, including animals, fungi, plants, and bacteria. In this section we report on our experience using the tool for this application. We follow with a formal evaluation by two structural biologists, expanded from our IEEE BioVis Data Contest Visualization Award-winning submission. We last report the feedback from the contest organizers.

### TIM protein-family exploration

The application examines the scTIM protein (saccharomyces cerevisiae triosephosphate isomerase), a member of the TIM family that was mutated towards the family consensus: a number of amino acids in the sequence were replaced by the most common residue found at that location in the TIM family. The resulting amino acid sequence is dTIM. Unfortunately, dTIM is functionally defective--one or more of the modifications made to scTIM caused the protein to lose its metabolic transport properties. Identifying which modifications caused the loss of functionality is an interesting open research problem.

For this application, we obtained the scTIM PDB, the TIM family sequence data and alignment information from the Battelle Center for Mathematical Medicine, through http://www.biovis.net. We used the tool to fetch 28 additional PDB files from RCSB, and to further generate more than 620 PDB files from the provided sequence data. We used the database backend to link PDB and FASTA IDs for preprocessing, and added data from ModBase and Uniprot.

**Figure 4:**
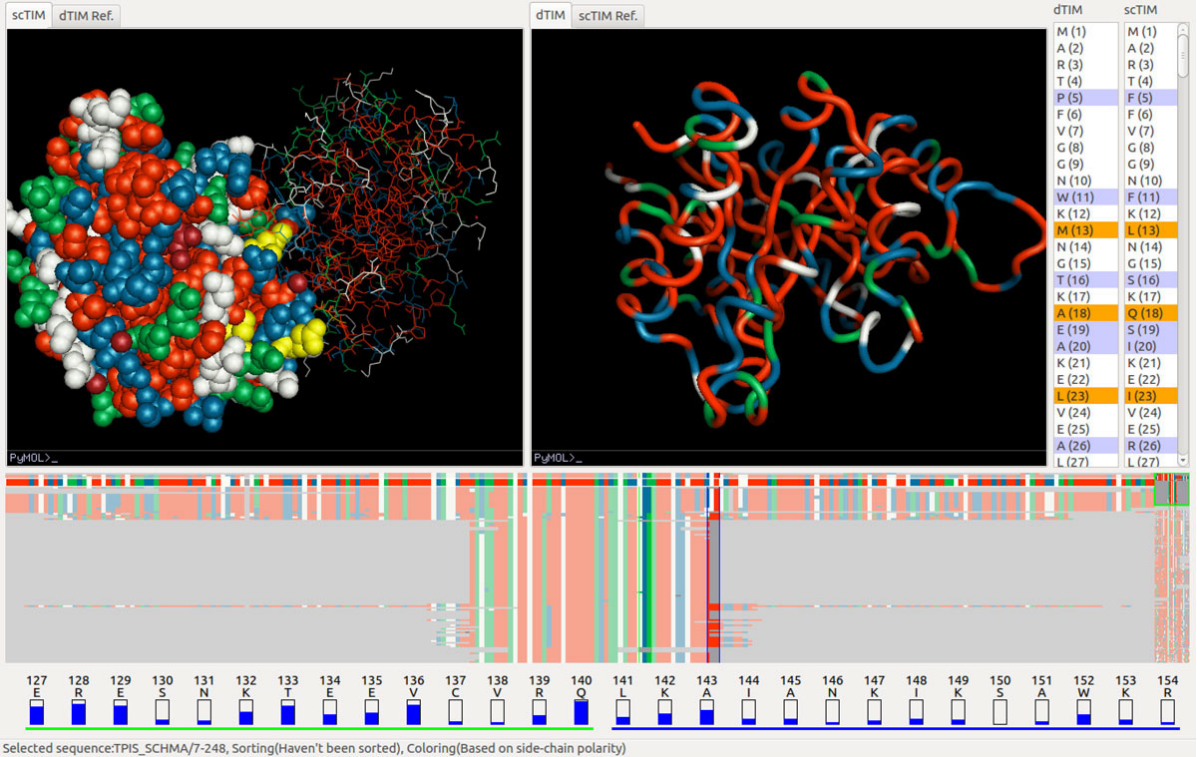
**scTIM/dTIM comparison**. The two volume views show the dTIM protein backbone (right), respectively a CPK sphere representation of scTIM (left). The trend image is sorted by the number of common residues. A side-chain polarity coloring is applied, and the two vertical selection paddles are located around position 142. The two residues shown at the bottom (green underline, respectively blue underline) correspond to the two vertical paddle selections.

Using our tool, we start by identifying the differences between the dTIM and scTIM sequences. There are 49 different subsequences of residues, encompassing 104 residues modified, created, or deleted in the creation of dTIM. By selecting some or all of these residues in the protein sequence viewer, we can highlight their locations on both 3D structures (Figure 4). We can pan, zoom, and rotate the structures to more closely examine the distribution of these alterations on the protein structure. We can also adjust the rendering properties of the structure.

To determine which models from the TIM family are most similar to the original scTIM, we use the trend-image view in the lower panel. In Figure 4 we can quickly see, for example, that only a few sequences have the same fragment in position 142 with scTIM. A step further, selecting any of the sorting modes from the menu allows comparisons to be made to scTIM. For example, when sorting by common residues, we find that TPIS HAEDU, the TIM protein homolog found in bacterial species *Haemophilus ducreyi*, shares the greatest number of residues with scTIM. Selecting a particular coloring method displays specific information for each residue.

Manipulating the vertical selection paddle allows us to explore subsequences of the full TIM sequence. Distribution information about residues in the highlighted subsequences are displayed below the trend image and show the most common amino acid in the TIM family at each sequence index. The bars in the residue viewer that are nearly empty imply that very few members of the TIM family share the same residue as scTIM, making it an ideal candidate for mutation towards the family consensus.

Manipulating the horizontal selection paddle allows us to further explore the individual TIMs in the family, with a fish-eye lens expanding the selected row to more clearly show the residue sequence and coloring. Right-clicking on a selected row allows us to load the structure of that specific TIM into the structure view. If this TIM is unfamiliar to the user, a number of reference databases can be accessed.

In terms of limitations, while the trend image provides a scalable approach to viewing large amounts of sequence data, finding a particular sequence in a protein family remains a challenge. Similarly, attempting to code too much information into the color schemes results in an overload of colors, rendering the trend image unreadable and ineffective. A reduction in the number of colors restores readability to the view, at the cost of removing some information from the trend image.

### Structural biologist feedback

Two senior structural biology researchers (co-authors DK and TT) have provided feedback and testing throughout the software development process. They are also providing the following example workflow through our system.

In this evaluation session, the researchers sought to explore the mutations in the BioVis Data Contest dataset. Given their structural biology background, the researchers began their analysis by loading and interacting with the 3D structures of the dTIM and scTIM proteins. Their interaction focused on searching for the residues that make up the active site and the protein-protein interface. In their estimation, these two sites were likely candidates for the location of functional mutations.

**Figure 5:**
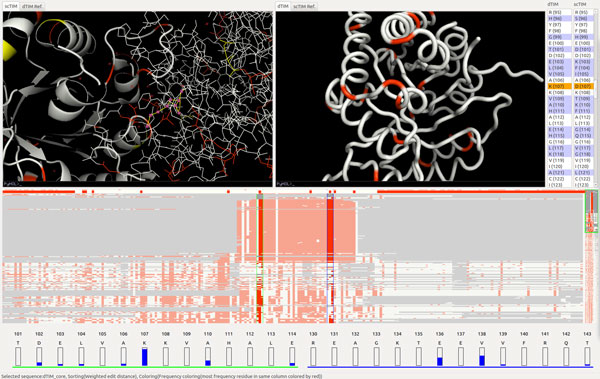
**Two residue mutations that may restore function to the protein; residues (K)107(D) and (E)138(E)**. For both residues, dTIM shares the family consensus but differs from its parent, scTIM.

The researchers selected next the key residues in the 3D Viewer. This action highlights those residues in both the Trend Image Panel and the Protein Sequence Viewer, as shown in Figure 5. Using the Protein Sequence Viewer, the researchers identified which of these residues had changed in the conversion from scTIM to dTIM. For each different residue, the researchers returned to the 3D Viewer to inspect the structure of each of the residues and examine their interactions. At this stage the researchers did not use the remaining genomic data, as they were unsure of how to best use this information for the purpose of the contest. Under these circumstances, they identified and proposed two relevant mutations as the most likely candidates: Y101 (to E100) and D81 (to P80).

However, by using the trend image panel, the researchers were able to identify several further matching sequences in the trend image. Although during the evaluation session the trend alignment and residue numbering within a sequence were slightly off due to insertions and deletions in the sequence (later accounted for and corrected in the software), the most senior researcher was able to identify the same set of candidate mutations as captured in the case study above.

In the researchers' assessment, it "*would be possible to come up with some reasonable hypotheses without using [this] tool, but it would definitely take more time." *In the default workflow, the researchers believe they would start by building a homology model for dTIM using Modeller and then align this model with the known structure for scTIM within PyMOL, followed by proposing a list of mutations that could be relevant, for example those localized to the active site. However, this approach would not leverage the information of the protein sequence family. To access this type of information without our tool, one could create a multiple sequence alignment using any of a number of online servers, then load the result in an alignment viewer such as JalView [12], and then go back and forth between the structures in PyMOL and the alignment viewer, in order to refine the previous list of mutations. However, in the researchers' opinion, this alternative approach would be tedious. As such, they particularly appreciated our tool's integration of the capabilities of existing spatial and sequence viewers, along with other useful functionality built within our software, and the speedup to such workflows provided by our approach.

### BioVis contest organizer feedback

Feedback from the BioVis 2013 conference organizers further confirmed the ability of our tool to successfully identify the dysfunctional protein mutations. The experts hypothesized that the most harmful mutations to the protein existed in the active site--the area of the protein which is responsible for its function. Since dTIM was created by combining the mutations of its 640 family proteins, the Trend Image Panel was first observed by the experts in order to gauge the difference of scTIM to the rest of its family. When sorted by the weighted edit distance between scTIM and its protein family members, the trend image exposed five distinct residue locations where dTIM varied from scTIM, but was consistent with the rest of its family. These locations are in our assessment (A)58(G), (K)107(D), (E)138(L), (L)146(V), and (A)22(R) and (L)218(V). From these mutations, the 107, 138 and 146 residues are almost fully conserved throughout the entire family, but differ in scTIM to dTIM. While residue 138 looks promising, since it is very frequent across the entire family, closer inspection shows that a mutation did not occur between dTIM and scTIM. In contrast, mutation 58 is also highly conserved throughout the TIM family, but is also a mutation from scTIM to dTIM. Finally, the last two mutations are both symmetrical in position, at an offset of 22 from either end of the sequence; these residues are also highly conserved in the TIM family, but not between scTIM and dTIM, which indicates they are not responsible for the loss of functionality.

Further examining the 3D structure of the four remaining residues (excluding the two symmetric ones) from the candidate list above, we notice that they lie in or just outside of the active site. Again, this is where most chemical reactions occur, since the active site is the binding site of molecules. This observation brings us full circle to the earlier structural biologist feedback: the structural biology experts initially suspected the most damaging mutations would lie in the active site, and that restoring these mutations could restore functionality.

In terms of tool features, the trend image and its sorting capabilities--based on our proposed metrics of similarity--were greatly appreciated by the IEEE BioVis domain experts evaluating the tool. A closer examination through the use of the 3D Model Viewer provided evidence that the residues identified above were part of the active site of the protein. This finding matched the initial hypothesis of where the most critical mutations existed, and demonstrates the benefits of combining spatial and non-spatial information in a single tool.

Without the aid of our tool, each sequence would have to be collected and aligned to determine the areas of residue variance. Once found, individual models of dTIM and scTIM have to be examined to locate the area where the mutations were occurring on the 3D structure. By using our tool, the experts were able to quickly identify the residue mutations and link them to the 3D model with a single action. Each expert biologist that tested our tool noted the ease in interaction between the different data representations--sequence alignment, model interaction, etc. These features, coupled via the linked data views, proved very efficient for the task of identifying protein mutations. With the insights gained, the locations of where the sequence needs to be repaired were quickly identified.

## Conclusions

The TIM application shows that our tool can assist in the navigation of family of proteins, as well as in the exploration of individual protein structures. The side-by-side 3D views facilitate visual comparison, while the trend image abstraction provides an effective view and exploration of large collections of sequence data. Our tool integrates successfully multiple sources of information and both spatial and non-spatial data. Furthermore, a computational backbone facilitates sorting collections of sequences, as well as generates 3D structures for modified sequences.

In conclusion, we introduced a novel visualization tool that integrates 3D structural information and sequence information for a protein, with additional information from the multiple sequence alignment of the family of proteins with the same function, and with meta information extracted from the family data. In conjunction with domain expert knowledge, this interactive tool can help provide biophysical insight into why specific mutations affect function, and potentially suggest additional modifications to the protein that could be used to rescue functionality.

## List of abbreviations used

TIM: *triosephosphate isomerase*, scTIM: *saccharomyces cerevisiae triosephosphate isomerase*, dTIM: defective triosephosphate isomerase, PDB: protein data bank.

## Competing interests

The authors declare that they have no competing interests.

## Authors' contributions

TL contributes to this work the design and implementation of the client-server architecture, the database end, the 3D view and trend image of the visual interface. JW contributes to this work the sorting algorithms for the trend image, and several of the data parsers. KC contributes the design and implementation of the sequence and residue views. DK and TT are equal contributors to the testing of the software, and are co-authors of the reported feedback and case study. GEM conceived this project, contributed the top-level design and directed the design, implementation and testing of the tool. TL, KC, JW, DK, TT, and GEM contributed to the manuscript.

## Declarations

Publication costs were supported by grant NSF-IIS-0952720.

This article has been published as part of *BMC Proceedings *Volume 8 Supplement 2, 2014: Proceedings of the 3rd Annual Symposium on Biological Data Visualization: Data Analysis and Redesign Contests. The full contents of the supplement are available online at http://www.biomedcentral.com/bmcproc/supplements/8/S2
